# Indirect Genetic Effects on Alcohol Use Disorder and Nicotine Dependence

**DOI:** 10.64898/2026.04.17.26351089

**Published:** 2026-04-19

**Authors:** Mannan Luo, Victória Trindade Pons, Michael Zakharin, Jean-Baptiste Pingault, Nathan A. Gillespie, Hanna M. van Loo

**Affiliations:** 1 Department of Psychiatry, University Medical Center Groningen, University of Groningen, Groningen, the Netherlands; 2 Virginia Institute for Psychiatric and Behavioral Genetics, Virginia Commonwealth University, Richmond, VA, USA; 3 Department of Clinical, Educational and Health Psychology, Division of Psychology and Language Sciences, University College London, London, United Kingdom; 4 Social, Genetic and Developmental Psychiatry Centre, Institute of Psychiatry, Psychology and Neuroscience, King’s College London, London, United Kingdom

## Abstract

Substance use disorders run in families, yet the mechanisms underlying intergenerational transmission remain unclear. We investigated indirect genetic effects, pathways through which parental genotypes influence offspring phenotypes via the family environment, for alcohol use disorder (AUD), nicotine dependence (ND), and related quantitative outcomes, and aimed to identify family environmental factors through which such effects may operate. Using transmitted and non-transmitted polygenic scores (PGS) constructed for problematic alcohol use, tobacco use disorder, and general addiction liability, we analyzed 5972 European-ancestry adult offspring with at least one genotyped parent from the population-based Lifelines cohort (Netherlands). Offspring outcomes included lifetime DSM-5 AUD diagnosis, AUD symptom count, maximum drinks in 24 hours, Fagerström Test for Nicotine Dependence score, and cigarettes per day. AUD findings were meta-analyzed with data from the Brisbane Longitudinal Twin Study (*N* = 1368; Australia). We also examined parent-of-origin effects and mediation by parental substance use and socioeconomic status using structural equation modeling. Transmitted PGS robustly predicted all AUD and ND outcomes (*β* = 0.07–0.16; OR = 1.20 for AUD diagnosis). Non-transmitted PGS, indexing indirect genetic effects, were negligible for all clinical syndrome outcomes. The only significant indirect genetic effect was on cigarettes per day (*β* = 0.03, *p* = 0.01), mediated by parental smoking behavior but not socioeconomic status. These findings indicate that intergenerational transmission of risk for AUD and ND is driven primarily by direct genetic effects, with modest indirect genetic effects on smoking quantity. Larger samples and cross-trait analyses are needed to further elucidate these mechanisms.

## Introduction

Substance use disorders (SUDs), including alcohol use disorder (AUD) and nicotine dependence (ND), run in families [1–3]. Offspring of parents with SUDs have a nearly threefold increased risk of developing the same disorder [4, 5]. However, understanding how this risk transmits across generations remains challenging because parents provide both genes and rearing environments [2]. Many putatively environmental risk factors, such as parental substance use or socioeconomic status, are themselves genetically influenced [6]. This potentially induces gene-environment correlation, making it difficult to disentangle the extent to which intergenerational transmission operates through inherited DNA, the rearing environment, or their interplay [7].

One pathway through which gene-environment correlation arises is indirect genetic effects, whereby parental genotypes influence offspring outcomes through the environments parents provide [8, 9]. Although often described as genetic nurture (e.g., parenting processes within the family), indirect genetic effects can also shape offspring outcomes through broader pathways that do not directly involve within-family nurturing [10]. For example, parental genetic liability to AUD may contribute to heavier drinking that normalizes alcohol use in the home [11], or to functional impairment that reduces socioeconomic status and affects neighborhood selection [12], both of which could increase offspring AUD risk independently of parentally transmitted alleles. The transmitted/non-transmitted polygenic score (PGS) design leverages parent-offspring genetic data to separate these pathways by partitioning parental alleles into (i) transmitted PGS (PGS_T_), capturing the combined influence of direct and indirect genetic effects, and (ii) non-transmitted PGS (PGS_NT_), indexing indirect genetic effects independently of direct transmission [8, 9].

Previous studies, including our own, have detected modest indirect genetic effects on quantitative substance use outcomes such as cigarettes per day [13–15]. However, it remains unclear whether these effects generalize to clinically defined SUDs in the general population, specifically AUD and ND, two of the most prevalent and burdensome SUDs worldwide [16, 17]. To date, only one study (*N* = 4,806) has reported indirect genetic effects on AUD, in a high-risk sample enriched for familial alcohol problems [18]. It is also unknown whether such effects differ by parent-of-origin in clinical outcomes, as aggregating maternal and paternal PGS could obscure parent-specific direct [19] and indirect genetic effects [8]. Furthermore, parental substance use has been identified as a plausible mediating pathway for indirect genetic effects on offspring non-clinical outcomes such as smoking [13, 14], yet the mechanisms underlying such effects on clinical SUDs remain poorly understood. In particular, it is unclear whether they operate through parental substance use [20, 21] or socioeconomic disadvantage [22, 23], a transdiagnostic risk factor for SUDs.

To address these gaps, we constructed PGS_T_ and PGS_NT_ to investigate direct and indirect genetic effects on offspring AUD, ND, and related quantitative phenotypes. Primary analyses were conducted in the population-based Lifelines cohort (the Netherlands). Given that indirect genetic effects are typically small [13, 14, 24, 25], we additionally meta-analyzed Lifelines with the Brisbane Longitudinal Twin Study (BLTS, Australia) for AUD outcomes, the only phenotype with comparable diagnostic assessments across cohorts, to increase power and assess cross-population generalizability. This study aimed to: (i) examine whether parental genotypes show indirect genetic effects (indexed by PGS_NT_) on AUD and ND, using both substance-specific PGS and a general addiction liability PGS; (ii) evaluate whether maternal and paternal indirect genetic effects differ in magnitude; and (iii) elucidate whether these effects are mediated by parental substance use, socioeconomic status (parental education and household income), or both.

## Materials and methods

The study protocol was preregistered with the Open Science Framework (https://osf.io/xu9w7/); deviations are described in the Supplementary Methods.

## Participants

Primary analyses were conducted in the Lifelines cohort. Lifelines is a multi-disciplinary prospective population-based cohort study examining in a unique three-generation design, the health and health-related behaviors of 167729 persons living in the North of the Netherlands. It employs a broad range of investigative procedures in assessing the biomedical, socio-demographic, behavioral, physical and psychological factors which contribute to the health and disease of the general population, with a special focus on multi-morbidity and complex genetics. Data collection was accomplished across three assessments waves: the baseline (2007–2013), wave 2 (2014–2017), and wave 3 (2019–2023). Detailed information on the study design and sample characteristics has been described elsewhere [26, 27]. For the present study, we included 19233 genotyped adult offspring with at least one genotyped parent, comprising 15966 parent-offspring pairs (father-child or mother-child) and 3267 complete trios (mother-father-offspring). Analytic sample sizes varied by outcome availability, ranging from 2083 to 5972.

For the AUD meta-analysis, we additionally included 1368 adult offspring with at least one genotyped parent from the BLTS [28], yielding a combined meta-analytic sample of 6229 offspring (Lifelines: *n* = 4861; BLTS: *n* = 1368). All other analyses, including ND-related outcomes, parent-of-origin effects, and mediation analyses, were conducted in Lifelines only due to limited data availability in BLTS.

## Phenotypic Measures

### Alcohol use disorder

In Lifelines, lifetime AUD was assessed at wave 3 (2019–2023) using DSM-5 criteria among ever-drinkers who reported retrospectively on their period of heaviest use. Lifetime AUD diagnosis was defined as endorsement of at least two DSM-5 symptoms [29]. To handle missing items while preserving diagnostic certainty, we applied a conservative PhenX Toolkit algorithm (Supplementary Methods). We also examined two continuous AUD-related outcomes to capture dimensional variation and improve statistical power: AUD symptom count (AUDsx) and maximum alcoholic drinks consumed in 24 hours (MaxDrinks), a proxy for binge drinking propensity [18]. In BLTS, lifetime AUD was assessed via self-report questionnaire using the same DSM-5 criteria (Supplementary Methods).

### Nicotine dependence

Lifetime nicotine dependence was assessed at wave 3 using the Fagerström Test for Nicotine Dependence (FTND) [30] in ever-smokers meeting a lifetime exposure threshold for at least one product (≥100 cigarettes or e-cigarettes, ≥25 cigars, ≥50 cigarillos, or ≥40 pipe sessions). Participants reported retrospectively on their period of heaviest tobacco use (Supplementary Methods). Those not meeting any exposure threshold were not administered the FTND questionnaire. FTND scores range from 0 to 10, with higher scores indicating greater dependence. We included lifetime average cigarettes per day (CPD) assessed at baseline, as a complementary severity indicator that correlates strongly with FTND [31] and has been shown to index genetic liability to dependence [32].

### Parental socioeconomic status

Parental socioeconomic status was indexed by years of education and equivalized household income, assessed via self-report at baseline (2006–2013). Educational attainment was derived from participants’ highest obtained educational degree, converted to the corresponding number of years of education required to complete that degree, following previous research in Lifelines [33]. Monthly household income was equivalized by dividing the midpoint of each income category by the square root of household size, thereby accounting for differences in household composition. For interpretability, income was rescaled by dividing by 100, so that estimates reflect differences per 100-euro change in equivalized monthly household income at baseline.

## Genotyping and polygenic scores

Details on genotyping, quality control, imputation, inference of transmitted and non-transmitted alleles, and PGS construction are provided in the Supplementary Methods. Briefly, 79988 Lifelines participants were genotyped across three batches and imputed using the Haplotype Reference Consortium v1 panel. Analyses were restricted to participants of European ancestry to minimize population stratification.

We used Haplotype-based Inference of Non-Transmitted Alleles (HINTA) [33], a validated method that overcomes a key limitation of standard transmitted/non-transmitted PGS designs: the requirement for complete parent-offspring trios. By inferring non-transmitted alleles from haplotype comparisons and recombination patterns, HINTA can reliably reconstruct parental non-transmitted alleles even when only one parent is genotyped, achieving 99.8% concordance with standard trio-based pseudo-control phasing approach [34]. This enables accurate distinction of transmitted and non-transmitted alleles in both parent-offspring duos and complete trios, increasing sample size, statistical power, and the generalizability of indirect genetic effect estimates.

Transmitted (PGS_T_) and non-transmitted (PGS_NT_) polygenic scores were constructed using summary statistics from the largest independent genome-wide association studies (GWAS) of problematic alcohol use (PAU) [35], tobacco use disorder (TUD) [36], and a general addiction factor for substance use disorders (SUDs) derived from a multivariate GWAS of problematic alcohol use, problematic tobacco use, cannabis use disorder, and opioid use disorder [37]. Variant weights were estimated using LDpred2-auto [38] and applied in PLINK 1.9 [39] to construct PGSs based on the transmitted and non-transmitted allele datasets.

To maximize statistical power for estimating overall effects, maternal and paternal transmitted and non-transmitted PGS were summed into combined parental scores. The missing parental PGS_NT_ values among parent-offspring pairs were imputed using the mean of observed parents, following previous work [8, 33]. Parent-of-origin and mediation analyses used separate non-imputed maternal and paternal scores (PGS_T_m_, PGS_NT_m,_ PGS_T_f_, PGS_NT_f_), with missingness handled by full-information maximum likelihood (FIML) in *lavaan* [40]. All PGSs were residualized on the first ten genetic principal components and standardized within each genotyping array to control for population stratification and batch effects.

## Statistical analysis

All analyses were conducted in R version 4.2.1 [41].

### Overall indirect genetic effects on AUD and ND

We used mixed-effects regression models to examine associations between parental PGS_T_ and PGS_NT_ with five offspring outcomes, including AUD diagnosis, AUDsx, MaxDrinks, FTND sum score, and CPD. Continuous outcomes were analyzed using mixed-effects linear regression with *lmerTest* package [42], and dichotomous outcomes using mixed-effects logistic regression with *GLMMadaptive* [43]. Models were specified as: ${Outcome}_{i}=\beta_{1}PGS_{Ti}+\beta_{2}PGS_{NTi}+\beta_{3}Sex_{i}+\beta_{4}Age_{i}+1|FamilyID\;+e_{i}$. Family ID was included as a random effect (intercept) to account for sibling relatedness, with offspring sex and age as covariates. Consistent with Kong et al. [8], the direct genetic effect ($\beta_{\text{DGE}}$) was estimated as the difference between the transmitted and non-transmitted PGS effects (i.e., $\beta_{T}-\beta_{NT}$). Because ${PGS}_{T}$ captures both direct and indirect genetic effects, subtracting $\beta_{NT}$ isolates the direct genetic transmission component. To correct for multiple testing across the five correlated outcomes, the effective number of independent tests was estimated using Galwey’s method ($M_{\text{eff}}=4$) with *poolr* [44], and a Šidák-corrected significance threshold of *p* < .013 was applied to balance family-wise error rate control with statistical power [45].

### Meta-analysis on AUD

Given the modest sample size for clinical AUD in Lifelines and small effect sizes typically expected for indirect genetic effects, we meta-analyzed transmitted and non-transmitted PGS effects on AUD across Lifelines and BLTS. Regression models were fitted separately in each cohort using identical model specifications, as described above. Cohort-specific effect estimates and standard errors were combined using fixed-effects inverse-variance-weighted meta-analysis with *metafor* [46].

### Parent-of-Origin effects

Unlike the mixed-effects regressions above, which combined maternal and paternal PGS to maximize power, we next fitted structural equation models (SEMs) in *lavaan* [47] to estimate all four PGS components simultaneously (PGS_T_m_, PGS_NT_m_, PGS_T_f_, PGS_NT_f_). This enabled formal testing of differences between maternal and paternal effects (both transmitted and non-transmitted) using likelihood ratio tests (*lavTestLRT*), comparing models in which maternal and paternal paths were constrained to equality against unconstrained models. Models were estimated using FIML, incorporating all available data without listwise deletion or prior imputation [40]. Offspring sex and age were included as covariates, and family ID was specified as a clustering variable with robust standard errors to account for sibling relatedness. All four PGS components were modeled simultaneously with freely estimated covariances, partially accounting for correlations between maternal and paternal PGS induced by assortative mating (non-random mate selection based on phenotypic similarity), and thereby reducing upward bias in indirect genetic effect estimates, although residual bias from multigenerational assortative mating cannot be fully excluded [48, 49].

### Mediation analyses

To identify which environmental pathways mediated indirect genetic effects, we conducted SEM-based mediation analyses in *lavaan*, restricted to outcomes with significant indirect genetic effects. Parental substance use, parental education, and household income were examined as candidate mediators. Each mediator was initially evaluated in a separate single-mediator model to assess its contribution independently. Significant mediators were then entered into a joint parallel multiple-mediator model to estimate their unique contributions after mutual adjustment. Maternal and paternal pathways were modeled simultaneously to enable parent-specific comparisons, and assortative mating was partially accommodated as described above. Confidence intervals for mediation effects and maternal-paternal difference parameters were obtained using Monte Carlo simulation with *semTools* (2,000 replications) [50]. Statistical significance was inferred when the 95% confidence intervals excluded zero.

## Results

In Lifelines, the analytic sample comprised 5972 adult offspring (mean age = 42.5 years, SD = 10.4), of whom 64.1% were female. Among ever-smokers meeting a lifetime exposure threshold for at least one tobacco product (*n* = 2083), the mean FTND score was 2.11 (SD = 2.16), reflecting nicotine dependence severity during their period of heaviest use. Among all ever-smokers, mean lifetime average CPD was 10.24 (SD = 6.00). Among ever-drinkers, the mean AUD symptom count was 0.88 (SD = 1.31), and 20.6% met criteria for lifetime AUD diagnosis (≥2 symptoms). Sample characteristics did not differ substantially between parent-offspring pairs and complete trios (Table 1). Bivariate correlations among PGSs and offspring outcomes are reported in the Supplementary Tables 1 and 2.

### Indirect genetic effects on AUD and ND

We examined associations of transmitted (PGS_T_) and non-transmitted (PGS_NT_) polygenic scores with offspring AUD- and ND-related outcomes, including clinical outcomes (lifetime AUD diagnosis and AUD symptom count) and quantitative measures (MaxDrinks, FTND sum score, and CPD) (Table 2).

Transmitted PGS for both substance-specific (PGS _t_PAU_, PGS_T_TUD_) and general addiction PGS (PGS_T_SUD_) showed robust associations with corresponding outcomes (*β*_T_ = .061–.161 for continuous traits; OR = 1.113 – 1.196 for AUD diagnosis; all p<.001). In contrast, indirect genetic effects, indexed by PGS_NT_, were largely absent. Of all associations tested, only one survived multiple-testing correction: parental PGS_NT_TUD_ was modestly associated with offspring CPD (*β*_NT_ = .033, p=.012). No other significant indirect genetic effects were observed for AUD diagnosis, AUDsx, MaxDrinks, or FTND sum score.

Direct genetic effects were quantified as β_DGE_ = β_T_ − β_NT_, following Kong et al. [8]. These effects were most pronounced for FTND sum score (β_DGE_ = .130) and CPD (β_DGE_ = .094) using PGS_TUD_. The sole significant indirect genetic effect, parental PGS_NT_TUD_ on offspring CPD, was approximately 35% of the corresponding direct genetic effect.

### Meta-analysis of AUD

**Figure 1.**
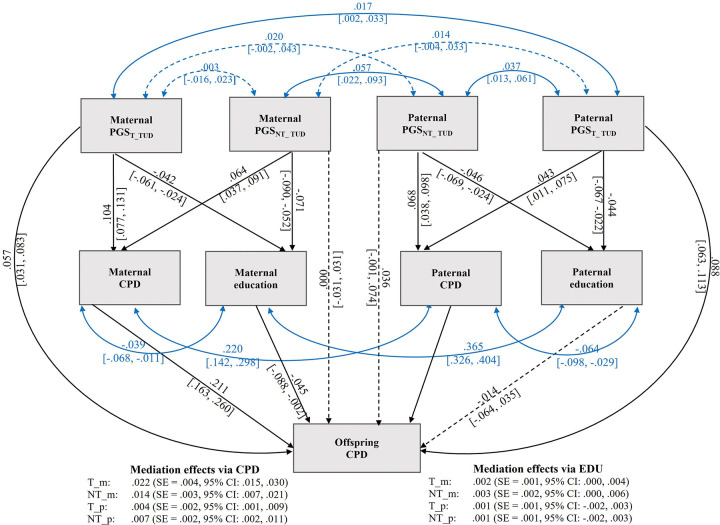
Parallel mediation model of offspring smoking quantity via parental smoking and parental education *Note.* Structural equation model testing maternal and paternal smoking quantity (CPD) and parental education as parallel mediators of associations between (PGS_T_TUD_) and non-transmitted (PGS_NT_TUD_) polygenic scores and offspring CPD. Models were estimated using full information maximum likelihood (FIML) with cluster-robust standard errors, adjusting for offspring sex and age. The total analytic sample passed to the model was *N* = 19,233. Observed Ns varied across variables due to data availability: PGS_T_ = 19,233 (both parents); PGS_NT_ = 13,236 (maternal) and 9,264 (paternal); parental CPD *N* = 6,472 (maternal) and 5,152 (paternal); parental education *N* = 12,965 (maternal) and 9,095 (paternal); offspring CPD *N* = 5,972. Solid paths indicate associations with 95% Monte Carlo confidence intervals excluding zero; dashed paths indicate nonsignificant associations. Double-headed arrows denote freely estimated covariances between maternal and paternal PGS and parental phenotypes, partially accounting for assortative mating between parents. All path coefficients are standardized.

To enhance statistical power and assess cross-population generalizability, PGS_T_ and PGS_NT_ effects on AUD were meta-analyzed across Lifelines and BLTS (total *N* = 6229; Supplementary Table 3 and Figure 1). Indirect genetic effects remained nonsignificant for both PGS_NT_PAU_ and PGS_NT_SUD_, consistent with Lifelines findings.

### Parent-of-origin effects

To examine whether maternal and paternal indirect genetic effects differed, transmitted and non-transmitted PGS were decomposed by parent-of-origin (Table 3). Both maternal and paternal PGS_T_ significantly predicted all offspring outcomes, with comparable effect sizes across parents (e.g., PGS _T_TUD_ on FTND: *β*_maternal_ =.104; *β*_paternal_ = .119). For indirect genetic effects, only paternal PGS_NT_TUD_ showed a nominal association with offspring CPD (*β* = .046, *p* = .017), which did not survive correction for multiple testing. No parent-specific indirect genetic effects were observed for ND or AUD outcomes using PGS_NT_SUD_. Likelihood ratio tests indicated no significant maternal–paternal differences in either transmitted or non-transmitted PGS effects for any outcome (all *p* > .25).

### Mediation analysis

Given that significant indirect genetic effects were observed only for CPD, we examined whether these effects operated through parental smoking, parental education, and household income (Supplementary Table 4). In single-mediator models, parental smoking emerged as the primary mediator, with maternal PGS_NT_TUD_ showing stronger mediation via maternal smoking (*β* = .014, 95% MC CI [.007, .021]) and paternal PGS_NT_TUD_ via paternal smoking (*β* = .007 [.002, .011]). Significant mediation was also observed for transmitted PGS_T_TUD_ via maternal smoking (*β* = .022 [.015, .030]) and paternal smoking (*β* = .004 [.001, .009]). Maternal education showed weaker evidence of mediation for PGS_NT_TUD_ (*β* = .004 [.001, .007]) but no significant mediation via paternal education. Household income did not mediate effects for either parent.

To assess whether maternal education represented an independent mechanism or was confounded by parental smoking, we fitted a parallel multiple-mediator model including both mediators simultaneously (Figure 1). Parental smoking estimates were unchanged, whereas mediation via parental education was attenuated to non-significance after adjustment for smoking. The transmitted mediation effects (PGS_T_TUD_) via maternal smoking were significantly larger than the corresponding paternal effects (*β*_diff_ = .018 [.009, .027]); no other maternal-paternal differences were significant.

## Discussion

This study provides the first comprehensive population-based examination of indirect genetic effects on AUD and ND. Using transmitted and non-transmitted polygenic scores in 5972 parent-offspring pairs and trios, supplemented by a meta-analysis of AUD across cohorts (total *N* = 6229), we found that intergenerational transmission of AUD and ND occurs primarily through direct genetic effects. Indirect genetic effects were largely absent, with the exception of offspring smoking quantity (CPD). Mediation analyses suggested parental smoking as a plausible environmentally-mediated pathway, with minimal contribution from parental socioeconomic factors.

Our null findings for indirect genetic effects on AUD contrast with prior evidence from Thomas et al. [18], who reported such effects on AUD in a high-risk sample enriched for familial alcohol problems. This discrepancy may reflect methodological differences. Thomas and colleagues did not test the total effect of non-transmitted parental PGS on offspring AUD, the standard parameter for indirect genetic effects [8, 9], but instead examined specific mediated pathways (e.g., parental relationship discord). Such mediated effects are difficult to interpret without a significant overall PGS_NT_ effect, as individual pathways can reach significance even when total indirect genetic effects does not [51]. Moreover, these mediator-outcome associations remain observational and susceptible to genetic confounding, so apparent mediation may arise spuriously in the absence of true indirect genetic effects [52]. Finally, their high-risk sample likely captured more extreme environmental adversity, thereby increasing sensitivity to detect indirect genetic effects relative to population-based cohorts.

We observed divergent findings across nicotine outcomes: indirect genetic effects were detectable for lifetime average smoking quantity (CPD) but not for nicotine dependence (FTND). One explanation may be limited statistical power, as the FTND was assessed only at Wave 3 among a subset of ever-smokers meeting stricter inclusion criteria, reducing power to detect small indirect genetic effects relative to the broader CPD sample. Alternatively, this pattern may suggest that CPD and FTND capture distinct dimensions of nicotine-related risk. CPD quantifies cumulative smoking behavior across the lifespan and may be more sensitive to environmental influences such as parental behavioral modeling and household smoking norms [53, 54], whereas FTND captures physiological dependence during the period of heaviest use and may more strongly reflect neurobiological mechanisms and direct genetic liability [32]. Larger studies with more comprehensive smoking phenotypes will be needed to determine whether indirect genetic effects differentially influence behavioral versus physiological dimensions of nicotine dependence

A key contribution of this study is the dissection of intergenerational pathways using joint mediation models that allow parental phenotypes to mediate both transmitted and non-transmitted genetic effects on offspring. Although parental genetic liability for SUDs has been proposed to reduce socioeconomic status and thereby increase offspring substance use risk [13, 55], we found that the indirect genetic effect on offspring CPD operated primarily through parental smoking quantity, while the contribution of parental education was fully attenuated when parental smoking was included in the same model. This is consistent with social learning theory, which posits that transmission may occur through behavioral modeling, whereby children observe and imitate parental behaviors [56]. These findings should be interpreted cautiously, as the pathway from parental phenotype to offspring outcome remains observational and may be confounded by unmeasured environmental factors and residual genetic liability not captured by the PGS [52]. They therefore represent an exploration of plausible environmentally-mediated pathways rather than definitive evidence of causal mechanisms.

This study has several strengths, including its large population-based design, the separation of transmitted and non-transmitted alleles in genotyped parent-offspring pairs and trios, the inclusion of both clinical syndromes and dimensional traits, and the cross-cohort meta-analysis for AUD. Furthermore, SEM-based mediation modeling enabled simultaneous evaluation of multiple environmental pathways while partially accounting for assortative mating. However, several limitations should be noted. First, statistical power was constrained by modest sample sizes for certain outcomes, particularly FTND, and by limited predictive performance of current SUD PGSs [57]. Transmitted PGS effects were modest (β = .07−.16), suggesting that these scores capture only a fraction of heritable variance. Together, these factors reduced our ability to detect more nuanced indirect genetic pathways, including parent-of-origin patterns and socioeconomic mediation. Second, parental household income was reported when offspring were already adults, limiting its validity as a proxy for financial resources during critical developmental periods. Third, in addition to genuine indirect genetic effects, PGS_NT_ may capture residual confounding from population stratification and assortative mating [52, 58]. Although we adjusted for population structure using principal components and partially accounted for assortative mating within the SEM, residual bias may remain. Finally, analyses were restricted to individuals of European ancestry, limiting generalizability to diverse populations.

## Conclusion

In this large-scale, population-based study, the intergenerational transmission of risk for AUD and ND appears to be driven primarily by direct genetic effects, with minimal evidence for indirect genetic effects. The exception was a modest effect on offspring smoking quantity, operating through parental smoking rather than broader socioeconomic pathways. However, the absence of detectable indirect genetic effects does not preclude their role in SUDs, as such effects may be subtle and require greater statistical power to detect. Future research combining larger samples, more refined SUD measures, and increasingly predictive polygenic scores will be essential to detect and characterize indirect genetic effects in intergenerational transmission of SUDs.

## Supplementary Material

Supplement 1

## Figures and Tables

**Table 1.** Descriptive characteristics of Lifelines participants

	All		Pairs		Trios		*p*
	*N*	M±SD or n (%)	*N*	M±SD or n (%)	*N*	M±SD or n (%)	

Age (years) at assessment	5989	42.48 ± 10.44	4956	42.93 ± 10.36	1033	40.33 ± 10.53	<.001
Sex, female	5989	3838 (64.1%)	4956	3206 (64.7%)	1033	632 (61.2%)	.036
**Alcohol use disorder**							
AUD diagnosis, yes	4861	1002 (20.61%)	4009	827 (20.6%)	852	175 (20.5%)	.991
AUDsx	4862	0.88 ± 1.31	4009	0.88 ± 1.31	853	0.88 ± 1.28	.979
MaxDrinks	4762	12.67±7.47	3926	12.61 ± 7.43	836	12.95 ± 7.61	.237
**Nicotine dependence**							
FTND sum score	2083	2.11 ± 2.16	1762	2.15 ± 2.15	321	1.90 ± 2.19	.058
CPD*	5972	10.24 ± 6.00	5134	10.34 ± 6.06	838	9.59 ± 5.57	<.01

Note. *N* = Sample size, *M* = Mean, *SD* = Standard deviation. Chi-square test for binary outcomes and t-test for continuous outcomes when comparing the pairs versus trios on AUD- and ND-related outcomes. AUDsx =DSM-5 alcohol use disorder symptom count; MaxDrinks = maximum number of alcoholic drinks consumed in 24 hours; FTND = Fagerström Test for Nicotine Dependence; CPD = cigarettes per day;

* CPD was assessed at baseline. Sex assigned at birth was self-reported at baseline enrollment in Lifelines and used in all analyses.

**Table 2.** Associations of transmitted (PGS_T_) and non-transmitted (PGS_NT_) polygenic scores with offspring alcohol use disorder (AUD) and nicotine dependence (ND) outcomes in Lifelines

Predictors	Outcomes		PGS_T_			PGS_NT_			DGE
		*N*	*β*_T_ (SE)/OR_T_	95% CI	*p*	*β*_NT_ (SE)/OR_NT_	95% CI	*p*	*β*_DGE_

***Substance-specific PGS***									
PGS_PAU_	AUD diagnosis	4861	1.196	1.105, 1.294	<.001	0.959	.888, 1.035	.277	—
	AUDsx	4862	.078 (.014)	.050, .105	<.001	−.007 (.014)	−.035, .020	.598	.071
	MaxDrinks	4762	.068 (.012)	.044, .092	<.001	−.004 (.012)	−.028, .020	.721	.064
PGS_TUD_	FTND_sum_	2083	.161 (.022)	.118, .204	<.001	.031 (.022)	−.013, .074	.168	.130
	CPD	5972	.127 (.013)	.101, .152	<.001	.033 (.013)	.007, .059	.012	.094
***General addiction factor***									
PGS_SUD_	AUD diagnosis	4861	1.113	1.031, 1.202	.006	.975	.903, 1.053	.520	—
	AUDsx	4862	.061 (.014)	.033, .089	<.001	−.014 (.014)	−.042, .014	.322	.047
	MaxDrinks	4762	.068 (.012)	.044, .092	<.001	−.018 (.012)	−.042, .006	.140	.050
	FTND_sum_	2083	.121 (.022)	.077, .165	<.001	.0002 (.022)	−.043, .043	.990	.121
	CPD	5972	.116 (.013)	.091, .141	<.001	.021 (.013)	−.004, .046	.111	.095

Note. Mixed-effects regression models included up to 5,972 adult offspring with at least one genotyped parent and available outcome data. Family ID was included as a random intercept to account for sibling relatedness, with offspring sex and age as covariates. Continuous outcomes were analyzed using linear mixed-effects models and lifetime AUD diagnosis using mixed-effects logistic regression. PAU = problematic alcohol use; TUD = tobacco use disorder; SUD = substance use disorders; AUDsx = AUD symptom count; AUDsx =DSM-5 alcohol use disorder symptom count; MaxDrinks = maximum number of alcoholic drinks consumed in 24 hours; FTND = Fagerström Test for Nicotine Dependence; CPD = cigarettes per day; DGE = direct genetic effect. *β* = standardized regression coefficient; OR = odds ratio; SE = standard error; CI = 95% confidence interval. *p*-values are unadjusted. Šidák-corrected significance threshold: *p* < .013. β_DGE_ = β_T_ - β_NT_ for continuous outcomes.

**Table 3.** Parent-of-Origin effects on alcohol use disorder and nicotine dependence in Lifelines: transmitted and non-transmitted polygenic scores split by paternal and maternal haplotypes

	Maternal PGS_T_			Maternal PGS_NT_			Paternal PGS_T_			Paternal PGS_NT_		
	*β* (SE)	95% CI	*p*	*β* (SE)	95% CI	*p*	*β* (SE)	95% CI	*p*	*β* (SE)	95% CI	*p*

**Substance-specific PGS**												
***PGS_PAU_***												
AUDsx	.047 (.014)	.019, .076	.001	−.008 (.016)	−.041, .024	.618	.063 (.014)	.036, .089	<.001	−.007 (.021)	−.047, .033	.735
MaxDrinks	.044 (.012)	.021, .068	<.001	−.001 (.014)	−.028, .026	.929	.051 (.012)	.027, .074	<.001	−.017 (.018)	−.053, .019	.360
***PGS_TUD_***												
FTND_sum_	.104 (.022)	.062, .147	<.001	.007 (.026)	−.043, .058	.776	.119 (.022)	.077, .162	<.001	.053 (.034)	−.013, .119	.117
CPD	.078 (.013)	.053, .103	<.001	.016 (.016)	−.016, .047	.333	.095 (.013)	.070, .120	<.001	.046 (.019)	.008, .084	.017
**General addiction factor**												
***PGS_SUD_***												
AUDsx	.053 (.019)	.016, .091	.005	−.035 (.023)	−.081, .011	.142	.057 (.017)	.025, .090	.001	.007 (.028)	−.049, .062	.809
MaxDrinks	.055 (.012)	.032, .079	<.001	−.018 (.014)	−.045, .010	.203	.041 (.012)	.017, .065	.001	−.016 (.018)	−.052, .020	.378
FTND_sum_	.093 (.022)	.050, .135	<.001	−.025 (.025)	−.074, .023	.308	.078 (.023)	.032, .123	.001	.044 (.034)	−.024, .111	.204
CPD	.077 (.013)	.051, .103	<.001	.028 (.016)	−.002, .059	.071	.081 (.012)	.057, .105	<.001	.003 (.020)	−.036, .042	.879

Note. Structural equation models (SEMs) estimated maternal and paternal transmitted (PGS_T_) and non-transmitted (PGS_NT_) effects. Models were fitted in *lavaan* using full information maximum likelihood (FIML) with all available data from the genotyped family sample (parents and offspring; *N* = 19,233), with outcomes measured in adult offspring only; analytic sample sizes per outcome match Table 2.

Standardized coefficients (*β*), standard errors (SE), 95% confidence intervals (CIs), and unadjusted *p* values are reported. All models adjusted for offspring sex and age, and accounted for sibling relatedness using family ID as a clustering variable with robust standard errors. Because FIML for binary outcomes (AUD diagnosis) is not supported in lavaan, analyses were restricted to the four continuous outcomes. The Šidák-corrected significance threshold was *p* < .013.

## Data Availability

Individual-level data from the Lifelines Cohort are available under restricted access due to ethical requirements and privacy regulations protecting participant confidentiality. Researchers can apply to use the Lifelines data through the online application system. More information about how to request Lifelines data and the conditions of use can be found on their website (https://www.lifelines.nl/researcher/how-to-apply). GWAS summary statistics used to construct the polygenic scores were obtained from publicly available sources, including alcohol use disorder and substance use disorder GWAS from the Psychiatric Genomics Consortium (https://www.med.unc.edu/pgc/). Tobacco use disorder GWAS summary statistics are available upon request from the corresponding author (sanchezroige@ucsd.edu).
